# Effects of sleep quality on the offline storage state of visual working memory

**DOI:** 10.1186/s40359-026-05342-3

**Published:** 2026-08-10

**Authors:** Li Gong, Jinghan Chen, Xiaojing Li, Qiang Liu

**Affiliations:** 1 School of Education, Anyang Normal University, Anyang, 455000 Henan China; 2 Institute of Brain and Psychological Sciences, Sichuan Normal University, Chengdu, 610000 Sichuan China

**Keywords:** Visual working memory, Sleep quality, Online state, Offline state

## Abstract

**Objective:**

Visual working memory (VWM) is a core cognitive function in humans, which enables flexible and efficient storage of visual information from complex contexts into either online or offline states in accordance with current task demands. Among these two states, the offline state demonstrates greater advantages in resisting interference and stabilizing stored information. Previous research has demonstrated that an individual’s sleep quality can predict performance in VWM tasks. However, these studies have primarily focused on the online storage state of VWM. To date, there remains unclear whether sleep quality affects the offline storage state, and whether its influence differs between the two distinct storage states. This study aimed to clarify the behavioral association between sleep quality and VWM online and offline storage performance.

**Methods:**

The present study employed a mixed experimental design, combining the Pittsburgh Sleep Quality Index with a sequential presentation retrieval paradigm to examine the effects of sleep quality on different storage states of VWM. Valid data were obtained from 76 volunteers (mean age = 19.95 ± 1.72 years, male proportion = 27.63%). All participants completed the standard Pittsburgh Sleep Quality Index assessment to obtain comprehensive subjective sleep quality scores, which were adopted as the core predictive indicator for subsequent behavioral correlation analysis.

**Results:**

The experimental results showed that the total score of participants’ sleep quality was significantly negatively correlated with VWM accuracy in both online and offline states. In addition, for VWM accuracy, the main effects of sleep quality and VWM storage state were significant, and a significant interaction effect between sleep quality and storage state was also observed. Simple effect analyses further revealed that participants with higher sleep quality exhibited significantly higher VWM accuracy in the offline state compared with those with lower sleep quality. For VWM reaction time, only a significant main effect of VWM storage state was identified.

**Conclusions:**

These behavioral findings indicate that sleep quality exerts differential influences on distinct VWM storage states. Specifically, poor sleep quality imposes a more pronounced detrimental effect on VWM offline accuracy relative to the online state. This study provides empirical behavioral evidence supporting the differentiation between online and offline VWM storage states and supplements existing research on the association between sleep quality and VWM performance.

## Introduction

Visual working memory (VWM), as a critical subsystem of working memory, is specifically responsible for the temporary storage of visual information [1] and serves as the foundation for individuals to engage in higher-order cognitive activities such as decision-making and reasoning [2–4]. However, given that VWM capacity is inherently limited [5, 6], individuals must flexibly represent and store to-be-processed information in distinct states according to real-time task demands when confronted with complex multitasking situations. Consequently, researchers have proposed the multi-state model of working memory, positing that individuals prioritize the processing of items highly relevant to the current task by maintaining them in an online state, while storing items irrelevant to the current task but pertinent to upcoming tasks in an offline state [7–9].

Sleep is a core physiological basis for maintaining cognitive function and is crucial for offline memory consolidation and the functional regulation of brain regions. Impaired sleep quality can lead to neuronal damage, significantly disrupt information processing and resource integration within the brain, and further weaken the efficiency and stability of cognitive activities such as memory and attention [10, 11]. Existing studies have shown that individuals’ sleep status can predict their task performance in VWM [12–14]. It is important to clarify here that the present study focuses on habitual daily sleep quality measured via the Pittsburgh Sleep Quality Index (PSQI) [15], a subjective assessment of chronic sleep status. While distinct from experimentally induced acute sleep deprivation, sleep restriction or nap interventions, these sleep-related conditions share overlapping physiological and cognitive pathways, allowing cross-reference of relevant mechanistic findings. Accumulated evidence has confirmed that both chronic poor subjective sleep quality and acute sleep loss trigger overlapping neural impairments, including disrupted fronto-parietal control networks, impaired hippocampal synaptic plasticity, and weakened episodic memory consolidation [11, 16, 17]. Although these two types of sleep disturbance differ in duration and induction mode, they converge on identical core cognitive pathways linking sleep deficits to working memory decline. For example, Chee and Chuah [12] used the change detection paradigm to investigate the effect of sleep deprivation on VWM. Their results revealed that sleep deprivation impaired individuals’ VWM performance, and neuroimaging findings showed neural deactivation in the precuneus and the temporoparietal junction. In addition, researchers have examined the influence of daily sleep quality on VWM performance using the PSQI and various VWM experimental paradigms. Consistent findings indicate that individuals’ daily sleep quality significantly predicts VWM performance, such that individuals with poorer sleep quality exhibit worse VWM performance [14, 18]. However, most of these studies adopted simple memory probe tasks and only focused on how sleep quality affects the online storage state of VWM. To date, few studies have explored the effect of sleep quality on the offline storage state of VWM.

Research has established that the online and offline states of VWM constitute two dissociable and independent states [19–21]. On the one hand, at the level of cognitive performance, these two states operate at distinct time points and correspond to different task demands: the former is relevant to the current task, whereas the latter is irrelevant to the current task but pertinent to upcoming tasks [22]. On the other hand, from a neurobiological perspective, the maintenance of online-state items relies on neural activation induced by persistent neuronal firing, with core regulatory regions including the early visual cortex and frontoparietal cortex; by contrast, the neural activity of offline-state items cannot be directly detected through electrophysiological signals, and their mnemonic maintenance is primarily achieved via synaptic weight changes [20, 23, 24]. Relevant human neuroimaging evidence supports this dissociation, while two key electrophysiological observations originate from non-human primate experiments: Panichello & Buschman [24] recorded macaque single-neuron activity to verify synaptic-dependent offline storage, and Barbosa et al. [25] captured latent silent neural signals in non-human primate prefrontal circuits. Collectively, these human and primate findings further corroborate the notion that offline storage may operate through activity-silent mechanisms [8, 25]. Therefore, it can be hypothesized that sleep problems may exert differential effects on distinct VWM storage states.

Furthermore, by comparing and analyzing the storage characteristics and core properties of the offline state of working memory with those of episodic memory, Beukers et al. [26] explicitly proposed that the storage process of the offline state of working memory relies on the modulation of the episodic memory system. Existing studies have confirmed that the hippocampus serves as the core brain region underlying the processing and storage of episodic memory [27], and its functional integrity plays a decisive role in the normal operation of episodic memory. Using targeted transcranial magnetic stimulation (TMS) over neural networks, Hermiller et al. [28] precisely modulated the hippocampal-cortical network in humans. Combining behavioral measures and neuroimaging indices, their study confirmed a clear causal regulatory effect of functional connectivity between the hippocampus and cortical networks during episodic memory retrieval. On this basis, Chandra et al. [29] further constructed a refined neural circuit model of the neocortex-entorhinal cortex-hippocampus, and more clearly illustrated the core regulatory role of the hippocampus in the encoding, storage, and retrieval of episodic memory from the micro-level perspective of dynamic interactions within neural circuits.

Notably, abnormal sleep states exert pronounced adverse effects on the physiological functions of the hippocampus. Findings regarding hippocampal neuronal reactivation, memory replay and molecular signaling pathways below are primarily derived from rodent animal model studies [17, 29, 30]. It should be emphasized that these animal-based results only offer tentative mechanistic references rather than direct, definitive explanations for human cognitive performance, given the inherent gaps between rodent brain physiology and human cognitive behavior. Even so, such animal research remains valuable for generating testable mechanistic hypotheses to guide future human neuroimaging investigations. Sleep deprivation impairs the stability of molecular signaling pathways within the hippocampus, reduces neuronal reactivation and memory replay in hippocampal regions [17, 30], and consequently compromises hippocampus dependent cognitive functions such as episodic memory and associative memory. Regarding the phase-specific mechanisms of sleep, non-rapid eye movement (NREM) sleep dynamically modulates the efficiency of interregional information transfer by regulating neural communication patterns within the corticothalamic-hippocampal circuit, thereby influencing the consolidation and integration of memory information and ultimately enabling precise modulation of episodic memory. In contrast, sleep exerts distinct regulatory mechanisms on the online state of working memory, primarily by modulating neural activity within the frontoparietal inhibitory network to alter its cognitive control function, which further affects the maintenance and processing efficiency of online memory representations [31, 32]. Collectively, these findings systematically reveal divergent regulatory patterns of sleep across different memory types, indirectly indicating dissociable effects of sleep on distinct storage states of VWM. This provides critical theoretical support for further investigating how sleep quality modulates offline storage in VWM.

In summary, grounded on the aforementioned theoretical rationales and empirical evidence, this study hypothesizes that sleep quality exerts distinct effects on the online and offline storage states of VWM. Nevertheless, this hypothesis has not yet been systematically verified. Accordingly, the present study adopted the PSQI to assess individual sleep quality, and combined the change-detection task with the sequential presentation-retrieval paradigm to address this research question. In the sequential presentation-retrieval paradigm, memory arrays are presented sequentially. This design is based on the assumption that initially presented items shift to a passive state during the active maintenance of subsequently presented items [21, 33, 34]. Such an arrangement enables the coexistence of active and passive memory representations within a single working memory task. The present study aims to enrich empirical findings in the fields of sleep and VWM at the behavioral level, further advance the theoretical understanding of the multi-state model of working memory, and provide new behavioral empirical evidence to inspire follow-up research exploring the neural mechanisms underlying sleep-related cognitive functions. Furthermore, by clarifying the differential impacts of sleep quality on the online and offline states of VWM, this research offers critical theoretical support for explaining the central role of sleep in a broad range of cognitive processes such as learning and memory, and also lays a solid foundation for future relevant investigations.

## Materials and methods

### Participants

The sleep quality questionnaire used in the experiment was the Chinese version of the PSQI [15], revised by Liu and Tang [35]. This scale includes 18 items and uses a 4-level rating (0 ~ 3 points), with a total score range of 0 ~ 21 points. The higher the score, the poorer the sleep quality. During the preliminary screening phase of this study, 522 valid PSQI questionnaires were collected from undergraduate students at a university in the southwestern region of China (M ± SD = 5.70 ± 2.48). All 522 participants only completed the sleep quality scale along with demographic questions, and no additional health history questionnaires were administered throughout the screening process. The Cronbach’s α of the PSQI scale was 0.80. All participants who subsequently took part in the formal VWM experiment were newly recruited volunteers from this screening pool specifically for our experiment on sleep quality and VWM, rather than being selected from an existing laboratory database or other research projects. According to the validated Chinese clinical detection criteria established by Liu & Tang [35], participants with PSQI scores of 5 and below were categorized into the high sleep quality group, and those with scores of 8 and above were categorized into the low sleep quality group. Scores ranging from 6 to 7 reflect a mild intermediate sleep state that cannot be unambiguously allocated to either group. To prevent blurring the between-group manipulation and weakening the contrast between distinct sleep quality levels, we excluded all participants with intermediate PSQI scores of 6–7 from formal experimental recruitment. Eligible participants who volunteered for the subsequent experiment were scheduled accordingly.

Sample size was estimated a priori using G*Power 3.1 [36] for a 2 (between-subjects: sleep quality) × 2 (within-subjects: storage state) mixed repeated-measures analyses of variance (ANOVA). With a significance level of α = 0.01, a medium effect size of *f* = 0.25, and an anticipated statistical power of 95%, the total sample size needed was at least 76. A total of 78 undergraduate students were recruited for this experiment. Two students did not complete the entire experimental task. Ultimately, 76 valid data sets were obtained (38 in the high sleep quality group and 38 in the low sleep quality group). The participants had an average age of 19.95 ± 1.72 years, with 21 male students (27.63%). All participants had normal or corrected vision and no color blindness or color deficiency. This study received ethical approval from the Ethics Committee of Sichuan Normal University (Approval No: 2024LS06). All experimental procedures complied with the ethical principles of the Declaration of Helsinki. The experimental procedures were thoroughly explained to the participants, and their informed consent was obtained prior to the experiment. Participants were offered a cash reward upon completion of the experiment.

### Stimuli

The experimental paradigm was programmed using E-Prime 3.0 software (Psychology Software Tools, Pittsburgh, PA, USA). All stimuli were presented on a 23.8-inch LCD monitor with a refresh rate of 60 Hz and a resolution of 1920 × 1080 pixels. All participants sat on a chair located 70 cm away from the monitor. For the duration of the experiment, participants were asked to stare at a “+” fixation (with a size of 0.47 °) in the center of the screen. The stimuli consisted of colored squares (0.65° × 0.65°) presented on a gray background. The colors of all stimuli were randomly selected from ten distinguishable colors (blue, black, red, magenta, green, lime, cyan, white, yellow, purple) and were not repeated within a single trial. The experiment recorded participants’ accuracy (ACC) and reaction time (RT).

### Procedure

**Fig. 1:**
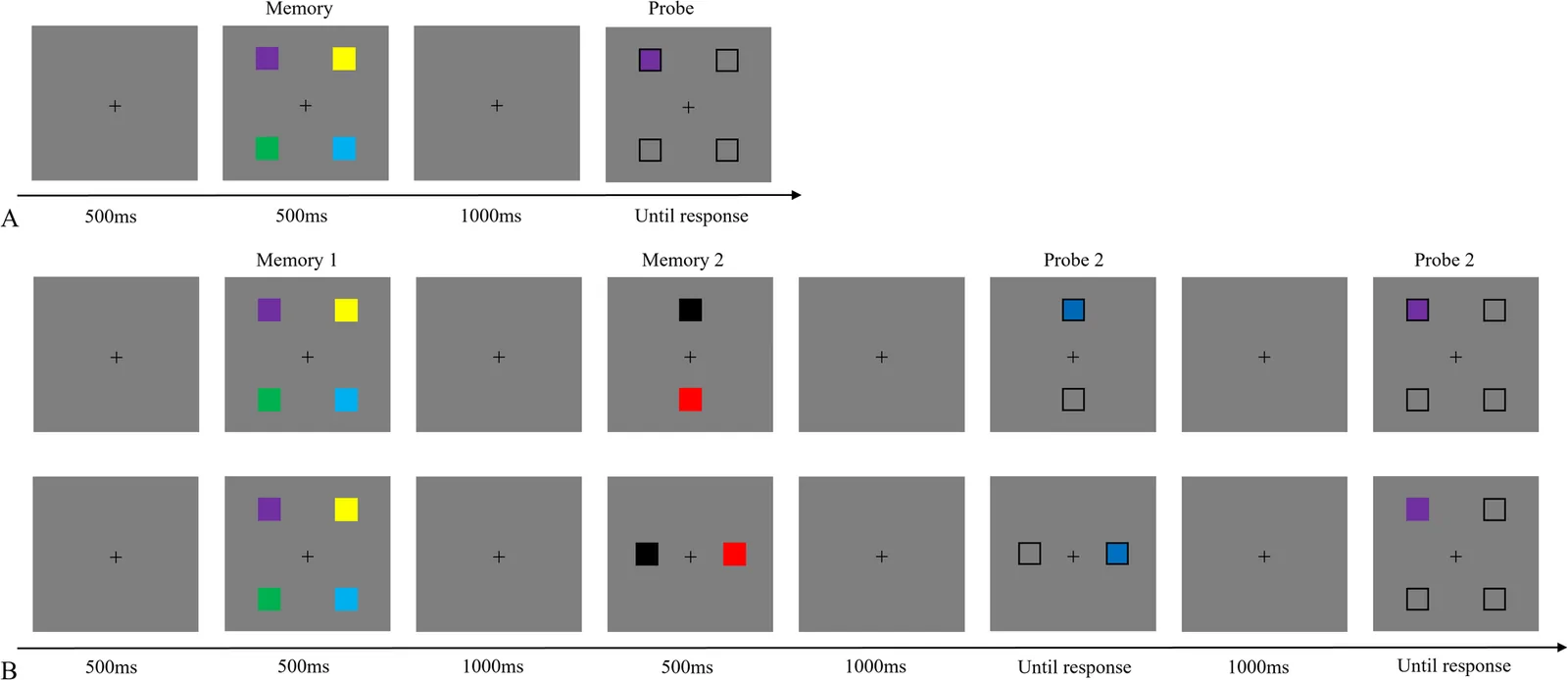
Experimental flowchart. **A** represents Task 1, and **B** represents Task 2

The experiment consisted of two tasks, and the order of these two experimental tasks was counterbalanced across participants. Task 1 (Fig. 1A) began with a blank screen lasting 500 ms, followed by a presentation phase where a memory array consisting of four items was presented for 500 ms. This was followed by a 1000 ms delay period. Finally, in the probe phase, participants were instructed to determine whether the detected item color was the same as the corresponding item color in the memory array. Task 2 (Fig. 1B) began each trial with a screen displaying a fixed cross, which lasted for 500 ms. Then, memory array 1 consisting of four items was presented for 500 ms. After a delay of 1000 ms, memory array 2 appeared, comprising two items. In half of the trials, these two items were presented vertically, while in the other half, they were presented horizontally. The order of these two conditions was counterbalanced across the experiment. Following the disappearance of memory array 2 and a delay of 1000 ms, the probe array appeared. First, participants were required to probe the items from memory array 2, followed by probing the items from memory array (1) To avoid confusion, we refer to the probing of memory array 1 as probe 1, and the probing of memory array 2 as probe (2) The squares appearing in probe 1 and probe 2 were consistent in terms of quantity and spatial location with the corresponding memory items from memory array 1 and memory array 2. During the probe phase, participants were instructed to judge whether the color of the probed item matched the color of the item at the corresponding position in the memory array. If they matched, participants were instructed to press the “Z” key; otherwise, they were instructed to press the “M” key.

We explicitly operationalized VWM online and offline states following established theoretical and experimental conventions [21, 33, 34]. The online state refers to real-time active maintenance of visual representations supported by sustained neural activity during stimulus encoding and immediate judgment, corresponding to Task 1. The offline state denotes activity-silent, latent memory storage that is disengaged from ongoing attention but retrievable for later testing. In Task 2’s sequential paradigm, the first memory array (tested after the presentation of the second array, Probe1) represents the offline state. Notably, Task 2 Probe 2 was only designed to distinguish dual memory states and was excluded from core analyses.

All participants received a cash reward upon completion of the entire experimental session. Each task began with 12 practice trials with real-time ACC feedback to help participants fully understand task rules and eliminate unfamiliarity-induced behavioral errors. The practice session served solely for task familiarization, and potential practice effects were minimized before formal data collection to ensure the validity of experimental results [16, 21]. Subsequent to the practice phase, the formal experimental procedure was conducted without trial-by-trial feedback. Each task included 3 formal blocks, and the whole experiment comprised a total of 96 formal experimental trials. The entire experimental procedure lasted approximately 35 min. Prior to the commencement of each block, a random four-digit number was presented on the screen, and participants were required to silently rehearse the number throughout the block to effectively suppress verbal encoding of visual stimulus information [21, 37]. All participants were instructed to complete the whole experiment with full concentration and rigorous compliance with task requirements.

### Statistical analysis

The data were analyzed using SPSS 27.0 (IBM Corporation, 2020), employing statistical techniques such as correlation analysis, two-factor repeated measures analysis of variance, and independent samples t-test. Normality was assessed with Shapiro-Wilk tests and Q-Q plots. RT is inherently right-skewed with a long tail; therefore, normality was evaluated mainly based on Q-Q plots and the absence of extreme outliers. All behavioral data were regarded as approximately normally distributed and suitable for parametric analyses. The correlation analysis was performed using a two-tailed Pearson test with the significance level set at α = 0.05. The significance level for all ANOVA was set at α = 0.05, two-tailed. Post-hoc simple effect comparisons after mixed ANOVA were adjusted with Bonferroni correction. In the change detection paradigm, the ACC measure not only assesses the precision of participants’ responses but also reflects their perceptual sensitivity and attention level. On the other hand, the RT measure provides a good indication of the speed and efficiency of participants’ cognitive processes. Therefore, in this experiment, the analysis focused on these two dependent variables: ACC and RT. We conducted single-trial outlier screening on each participant’s RT data. Any trial whose RT deviated more than ± 3 standard deviations from the participant’s own mean RT was discarded, and only the mean RTs of valid retained trials were analyzed.

## Results

### Descriptive statistics and correlation analysis of each variable

**Fig. 2:**
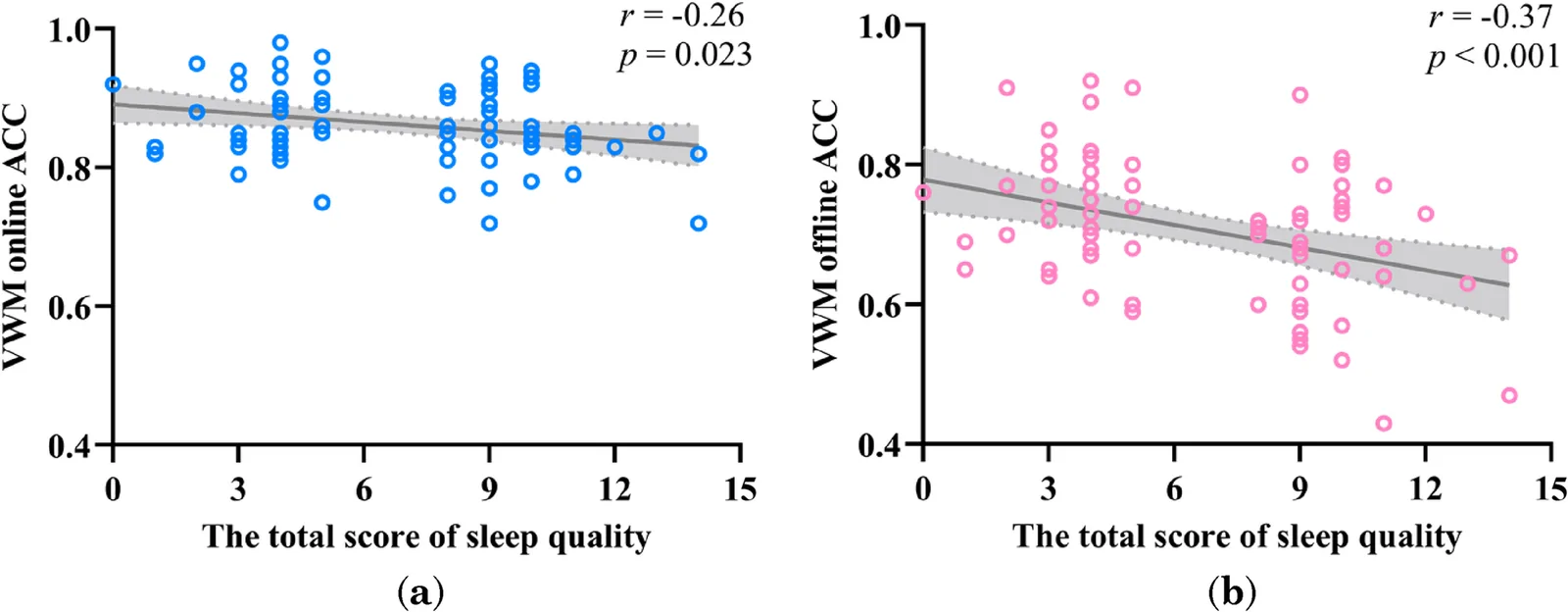
The scatter plot of correlation between total score of sleep quality and the ACC of online state (**a**) and offline state (**b**) of VWM

PSQI total scores were used as continuous variables in correlation analyses to examine the relationships between sleep quality and behavioral indicators (ACC and RT) of VWM in online and offline states. Notably, participants with intermediate PSQI scores ranging from 6 to 7 were excluded from formal testing to ensure sharp between-group differentiation for the primary mixed ANOVA analyses. As a result, the distribution of PSQI total scores in the current sample is truncated, lacking observations in the mild sleep impairment range. As shown in Table 1 below, the experimental results indicate that there was a significant negative correlation between the total score of sleep quality and the ACC of VWM in the online storage condition (*r* = − 0.26, *p* = 0.023). There was a significant negative correlation between the total score of sleep quality and the ACC in the offline storage condition (*r* = − 0.37, *p* < 0.001). However, there was no significant correlation between the total score of sleep quality of the students and the RT in different storage states of VWM. Furthermore, in Probe 2 of Task 2, sleep quality showed no significant correlations with either the ACC (*r* = − 0.16, *p* = 0.174) or RT (*r* = 0.16, *p* = 0.173) of VWM. Probe 2 was merely included to manipulate online/offline VWM dissociation rather than a primary outcome for testing our core hypothesis, so we focus analyses and discussion on offline memory storage. These results will not be elaborated upon further in the following sections. The scatter plot illustrating the correlation analysis between students’ total sleep quality scores and the ACC rates of two storage states in VWM is presented in the following Fig. 2.

**Table 1:** Means, standard deviations and correlation analysis among study variables (*N* = 76)

Variables	1	2	3	4	5
1. The total score of sleep quality	1				
2. VWM online ACC	−0.26^*^	1			
3. VWM offline ACC	−0.37^***^	0.57^***^	1		
4. VWM online RT	0.04	−0.04	0.03	1	
5. VWM offline RT	0.19	−0.14	0.05	0.78^***^	1
Mean	6.62	0.86	0.71	895.40	1347.60
SD	3.38	0.06	0.10	220.11	484.69

SD means standard deviation. ^*^ *p* < 0.05, ^***^ *p* < 0.001

### Repeated-measures ANOVA for sleep quality and VWM storage status

**Fig. 3:**
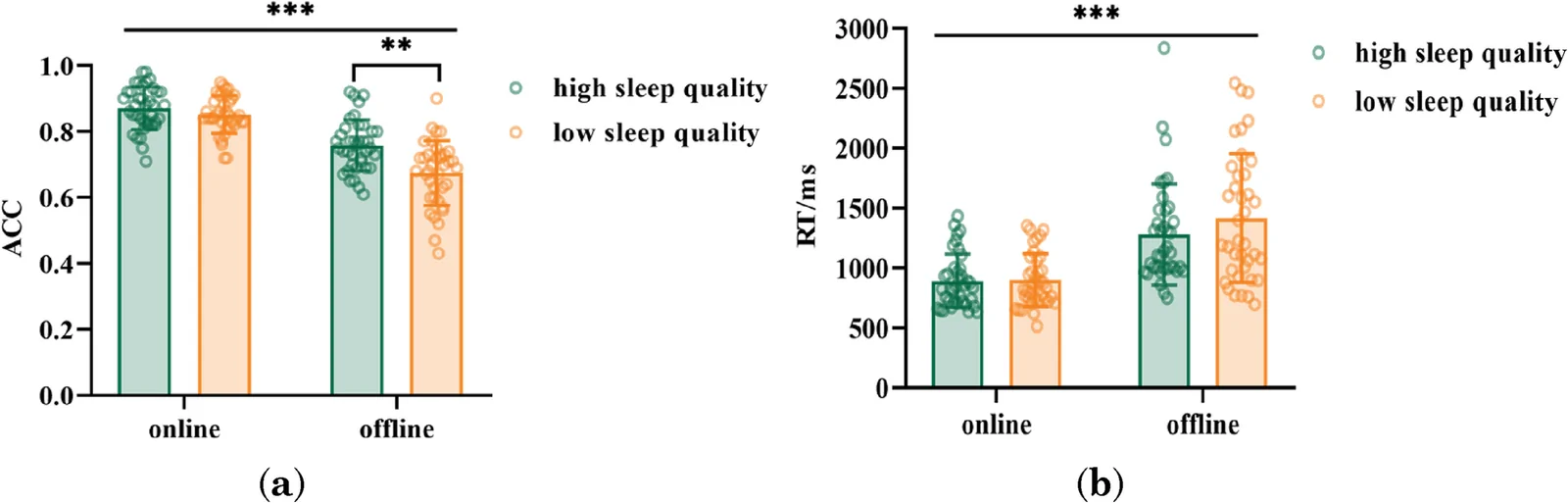
**a** ACC and (**b**) RT of online state and offline state under high and low sleep quality conditions. The error target in figure is standard error; ^**^ *p* < 0.01, ^***^ *p* < 0.001

Repeated-measures analysis of variance was further performed to explore the differences and interactive effects of VWM performance across different storage states between the high and low sleep quality groups. The ACC of VWM online and offline storage in high and low sleep quality groups was further analyzed by repeated measures analysis of variance. The results are shown in Fig. 3(a). The main effect of sleep quality was significant, *F* (1, 74) = 9.15, *p* = 0.003, *η*_*p*_^*2*^ = 0.11. The main effect of VWM storage state was significant, *F* (1, 74) = 303.47, *p* < 0.001, *η*_*p*_^*2*^ = 0.80. The interaction between sleep quality and VWM storage state was significant, *F* (1, 74) = 6.03, *p* = 0.016, *η*_*p*_^*2*^ = 0.08. Further simple effects analyses revealed that, in the online storage condition, the ACC difference between the two groups did not reach statistical significance, *t* (74) = 1.86, *p* = 0.067, Cohen’s *d* = 0.43, 95%CI [− 0.03, 0.88]. In the offline storage condition, the participants in the high sleep quality group (M ± SD = 0.74 ± 0.09) showed significantly higher ACC compared to the participants in the low sleep quality group (M ± SD = 0.67 ± 0.10), *t* (74) = 3.16, *p* = 0.002, Cohen’s *d* = 0.73, 95%CI [0.26, 1.19].

A repeated-measures analysis of variance was conducted to examine the RT of the high and low sleep quality groups in in the online and offline states of VWM. The results are shown in Fig. 3(b). The main effect of sleep quality was not significant, *F* (1, 74) = 0.87, *p* = 0.353, *η*_*p*_^*2*^ = 0.01. The main effect of VWM storage state was significant, *F* (1, 74) = 135.92, *p* < 0.001, *η*_*p*_^*2*^ = 0.65. The interaction between sleep quality and storage state was not significant, *F* (1, 74) = 2.86, *p* = 0.095, *η*_*p*_^*2*^ = 0.04.

## Discussion

This study employed a mixed experimental design to investigate the impact of sleep quality on different storage states of VWM by comparing the performance of high and low sleep quality groups on the online and offline states of VWM. The experimental results indicated that the main effects of sleep quality and VWM storage state were significant in the ACC index of VWM, the interaction between sleep quality and VWM storage state was also significant. Simple effect analyses demonstrated that in the offline storage state, the high sleep quality group exhibited significantly higher ACC compared to the low sleep quality group. Regarding the RT measures of VWM, there was a significant main effect of VWM storage state, while the main effect of sleep quality and the interaction between sleep quality and VWM storage state were not significant. These findings suggest that individual sleep quality has differential effects on different storage states of VWM, with a greater impact on the offline state.

The experiment reveals that the effect of sleep quality on the online and offline storage states of VWM is different, thus confirming our hypothesis. As mentioned above, these two distinct storage states of VWM differ qualitatively in both cognitive performance and neural mechanisms, and they do not share common storage resources [21]. The offline state relies on the episodic memory system, and the maintenance and retrieval of offline items require the involvement of the long‑term memory system [26, 38]. Specifically, existing studies have found that the neural decoding patterns underlying the offline state of working memory are largely consistent with those of episodic memory; both are maintained through rapidly formed changes in synaptic weights [26, 39]. Furthermore, Mızrak and Oberauer [40] adopted a modified Hebbian paradigm and serial recall task to investigate the contribution of long-term memory to working memory. Their findings indicated that the offline state of working memory selectively recruits task relevant information from the long-term memory system to optimize behavioral performance.

Accordingly, the mechanism underlying the influence of sleep quality on the offline storage of VWM may substantially overlap with that governing long-term memory function. Previous studies on sleep and memory have demonstrated that sleep quality exerts dissociable effects on the online storage of working memory versus long-term memory. Chen et al. [41] yielded relevant human behavioral and neuroimaging evidence, whereas Chernyshev et al. [42] and Guzeev et al. [43] verified related cognitive pathways at the molecular mechanistic level via rodent animal models. It is worth noting that the extant literature presents heterogeneous findings across different experimental designs, memory tasks and sleep manipulations. The following discussion is based primarily on evidence from human behavioral and neuroimaging studies, which are methodologically most comparable to our present paradigm. A body of research using declarative memory and episodic memory paradigms has documented that memory representations relying on long-term storage and synaptic consolidation are more susceptible to sleep disturbances [44, 45]. Conversely, several studies adopting classic working memory tasks with high online attentional demands found that poor sleep quality [16] and acute total sleep deprivation [46] each yield robust deficits in online working memory maintenance. Such discrepancies may stem from differences in task complexity, memory load, and the type of sleep manipulation adopted across studies. The present results align with the line of evidence observing heightened vulnerability in offline memory representations linked to the episodic memory system. These works provide convergent empirical support for the present research.

Furthermore, we found that sleep quality exerted a stronger influence on the offline state than on the online state of VWM. On the one hand, regarding the nature of the experimental paradigms, the online state is more analogous to the 1-back task, whereas the offline state resembles the 2-back task. Li et al. [47] investigated the effects of total sleep deprivation across varying working memory loads and found that performance on the 2-back task was impaired to a greater extent than performance on the 1-back task following sleep deprivation. On the other hand, prior studies have demonstrated that, under equivalent memory load conditions, behavioral performance is consistently worse in offline-state tasks than in online-state tasks. According to Zhang et al. [34], such behavioral patterns arise from memory loss occurring during the transfer of memorized items from the online to the offline state. This indicates that information maintained in the offline state is more limited and exhibits weaker memory traces compared with that retained in the online state. These findings are consistent with those reported by Peters et al. [48], who examined differences in information representation across VWM storage states and revealed that the feature precision of offline items is significantly lower than that of online items. Collectively, this evidence suggests that mnemonic strength in the offline state of working memory is weaker than that in the online state. Consequently, the offline state is more vulnerable to external factors such as sleep restriction.

Interestingly, some studies have found that sleep quality exerts a significant influence on performance in simple tasks requiring fewer cognitive resources. In contrast, such effects become non-significant during demanding, complex tasks that require intentional cognitive engagement [49, 50]. For instance, MacDonald et al. [13] focused on nap-related effects rather than total sleep deprivation, and highlighted the temporal variability of sleep-related effects on working memory. They proposed that the impairments associated with insufficient sleep are primarily driven by elevated fatigue. Notably, brief working memory tasks do not induce substantial fatigue in individuals. Instead, such tasks only require short periods of focused attention, which can offset the detrimental effects of poor sleep.

Unlike the online state of working memory, the offline state representation is stored through changes in synaptic weights. Synaptic connection strength, a critical component of neuronal communication, significantly influences brain function and the processing and storage of information [51]. The following accounts of synaptic plasticity are mainly supported by animal model research, which provides tentative mechanistic context rather than definitive causal explanations for the human behavioral patterns observed in this study. Research indicates that sleep deprivation or poor sleep quality can impair cognitive function by altering the number and morphology of dendritic spines and bidirectionally changing synaptic structural plasticity [52, 53]. The offline storage of VWM obviates the consumption of neural energy, thus not occupying excessive attentional resources during task performance [8]. Consequently, it can be integrated from the above that the impact of sleep quality on different storage states of VWM is dissociable, with a larger effect on the offline storage state than on the online storage state.

Our study investigated the effects of sleep quality on distinct storage states of VWM, with both theoretical and practical implications. First, this study is the first to explore the influence of sleep quality on the offline storage of VWM, thereby filling an important research gap in the relevant field. Second, it highlights the critical role of sleep in cognitive functioning and raises public awareness regarding sleep health. Third, our findings generate several novel research questions. For instance, as mentioned earlier, memory information is lost during transitions between different storage states. Does the effect of sleep quality on the offline storage of VWM originate from the cognitive costs incurred during memory switching between these distinct states? Furthermore, as reported by MacDonald et al. [13], nap-induced sleep modulation produces temporally variable effects on online working memory performance. Does such a phenomenon also manifest in behavioral performance related to the offline state? Finally, several limitations of the present study should be noted. First, this research only employed behavioral measurements, leaving the neural mechanisms underlying sleep quality modulation of VWM offline states unaddressed, which requires further neural-level exploration in future work. Second, the sample was demographically homogeneous, individual differences in occupation, and daily rest patterns were not systematically controlled, potentially confounding sleep and VWM performance. Third, sleep quality was assessed solely with the PSQI, a subjective scale. This method lacks objective sleep microstructural indicators and cannot establish precise quantitative links between objective sleep profiles and VWM offline states. Future research should incorporate objective sleep monitoring to address this limitation.

## Conclusions

This study employed a two-factor mixed experimental design to investigate the effects of sleep quality on different storage states of VWM. The results revealed that sleep quality exerted differential impacts on the online and offline states of VWM. Poor sleep quality was associated with greater impairment in the offline storage state. These findings provide new evidence for the relationship between sleep quality and VWM, and contribute to elucidating the inherent nature of their association.

## Data Availability

The data that support the findings of this study are available in the [figshare] repository (https://doi.org/10.6084/m9.figshare.25498384.v4).

## Abbreviations

VWM: Visual working memory
PSQI: Pittsburgh sleep quality index
ANOVA: Analyses of variance
ACC: Accuracy
RT: Reaction time
SD: Standard deviation
